# Bifunctional hydrous RuO_2_ nanocluster electrocatalyst embedded in carbon matrix for efficient and durable operation of rechargeable zinc–air batteries

**DOI:** 10.1038/s41598-017-07259-9

**Published:** 2017-08-02

**Authors:** Han-Saem Park, Eunyong Seo, Juchan Yang, Yeongdae Lee, Byeong-Su Kim, Hyun-Kon Song

**Affiliations:** 1 School of Energy and Chemical Engineering, UNIST, Ulsan, 44919 Korea; 2 Department of Chemistry, UNIST, Ulsan, 44919 Korea

## Abstract

Ruthenium oxide (RuO_2_) is the best oxygen evolution reaction (OER) electrocatalyst. Herein, we demonstrated that RuO_2_ can be also efficiently used as an oxygen reduction reaction (ORR) electrocatalyst, thereby serving as a bifunctional material for rechargeable Zn–air batteries. We found two forms of RuO_2_ (i.e. hydrous and anhydrous, respectively *h*-RuO_2_ and *ah*-RuO_2_) to show different ORR and OER electrocatalytic characteristics. Thus, *h*-RuO_2_ required large ORR overpotentials, although it completed the ORR via a 4e process. In contrast, *h*-RuO_2_ triggered the OER at lower overpotentials at the expense of showing very unstable electrocatalytic activity. To capitalize on the advantages of *h*-RuO_2_ while improving its drawbacks, we designed a unique structure (RuO_2_@C) where *h*-RuO_2_ nanoparticles were embedded in a carbon matrix. A double hydrophilic block copolymer-templated ruthenium precursor was transformed into RuO_2_ nanoparticles upon formation of the carbon matrix via annealing. The carbon matrix allowed overcoming the limitations of *h*-RuO_2_ by improving its poor conductivity and protecting the catalyst from dissolution during OER. The bifunctional RuO_2_@C catalyst demonstrated a very low potential gap (Δ*E*_OER-ORR_ = ca. 1.0 V) at 20 mA cm^−2^. The Zn||RuO_2_@C cell showed an excellent stability (i.e. no overpotential was observed after more than 40 h).

## Introduction

The demand for high energy-density technologies has gradually shifted the research interests from Li-ion to metal–air batteries^1–3^. The utilization of Zn in metal–air batteries is beneficial because of its low cost, the employment of aqueous electrolytes and the safety characteristics of this metal^4, 5^. However, a number of challenges lie ahead (i.e. non-uniform Zn dissolution from the anodes, limited solubility of Zn ions in the electrolytes and serious overpotential during charge) before rechargeable Zn–air battery cells are developed. In this sense, the development of efficient and stable bifunctional catalysts for air electrodes is one of the most important issues for rechargeable Zn–air batteries. The efficiency and cyclability of the oxygen reduction and evolution reactions (ORR and OER) should be guaranteed^2, 3^.

While platinum and ruthenium oxide (RuO_2_) are known to be the best ORR and OER catalysts, respectively^6, 7^, these materials exclusively promote these reactions (i.e. the electroactivity of RuO_2_ towards the ORR is low as compared with that towards OER)^8^. From a bifunctional viewpoint, the use of ORR-active RuO_2_ is the best scenario because we are already sure of its superior OER activities. This study was motivated by previous studies on the supercapacitor applications of RuO_2_^9–11^. Amorphous hydrous RuO_2_ (RuO_2_·*x*H_2_O with *x* > 0, *h-*RuO_2_) showed higher capacitances than its crystalline anhydrous counterpart (*x* = 0 or *ah*-RuO_2_)^10, 11^. This higher capacitance was potentially ascribed to a more active interaction between the space charges on the surface of *h-*RuO_2_ and the electrolyte ions, although this phenomenon does not provide a direct measure of the electroactivity. Despite its capacitance characteristics, *h-*RuO_2_ has not been proposed as an electrocatalyst yet, and studies describing the effect of the hydration on the electroactivity of this material are lacking in the literature. Therefore, the null hypothesis that partial or complete hydration of RuO_2_ is ineffective in improving the ORR and/or OER electroactivities is worth testing.

In case the null hypothesis is rejected, there is an additional issue. While *ah*-RuO_2_ shows high metallic conductivity (ca. 10^4^ S cm^−1^) and crystallinity, *h-*RuO_2_ has low electric conductivity (ca. 1 S cm^−1^) and amorphous characteristics^12^. The poor electric conductivity of the *h-*RuO_2_ catalyst particles is likely limiting their electrocatalytic activity. Three conditions are required to achieve high electrocatalytic activities: (1) rapid charge transfer kinetics^13^ defined by the catalytic active sites; (2) good accessibility of the reactants to the active sites^14^; and (3) the presence of highly developed electric pathways for the active sites^15^. Carbon coating of cathode and/or anode materials has been widely used as a key strategy to improve the electric conductivity of electrodes^16–18^. In this sense, electroactive materials can be easily composited with carbon by reducing the carbon precursors under a reductive gas environment at temperatures higher than the thermal decomposition temperature of the precursors and lower than the reduction temperature of the active materials. Therefore, the main concern when fabricating oxide/carbon composites via the thermal method is to prevent oxide reduction. For example, sucrose-coated Fe_2_O_3_ was converted to carbon-coated Fe_3_O_4_ at 500 °C under argon, and a third of the iron atoms were reduced from Fe^3+^ to Fe^2+^ as a result of the thermal treatment^19^.

Two points are crucial in our strategy to guarantee simultaneous bifunctional ORR and OER RuO_2_ electroactivities: (1) the presence of partially hydrated RuO_2_ as a catalyst and (2) carbon coating of the catalyst particles. We increased the electric conductivity of the catalyst layers by embedding RuO_2_ (or more precisely *h-*RuO_2_) nanoparticles in a carbon matrix phase (RuO_2_@C). The RuO_2_@C was synthesized by annealing micelles comprising RuO_2_ surrounded by double hydrophilic block copolymers of poly(ethylene oxide)-*block*-poly(acrylic acid) (PEO-*b*-PAA) as a template^9, 20, 21^. During the annealing process, the *h-*RuO_2_ core was crystallized while the PEO-*b*-PAA shell was converted to a continuous carbon phase surrounding partially hydrated RuO_2_. Both ORR and OER electroactivities were significantly improved by incorporating *h-*RuO_2_ into the carbon phase. Zn–air cells based on RuO_2_@C showed the very low potential gaps between the ORR (during discharge) and OER (during charge) processes, thereby confirming the superior reversibility of this material as compared with previously reported Zn–air cells utilizing Pt/C and *ah*-RuO_2_ catalysts.

## Methods

### RuO_2_@C Synthesis

Poly(ethylene oxide-block-acrylic acid) (PEO-*b*-PAA; PEO_5000_-*b*-PAA_6700_ where the numbers indicate molecular weights of each block; Polymer Source), ruthenium (III) chloride hydrate (RuCl_3_·*x*H_2_O; Sigma-Aldrich) and hydrazine (N_2_H_4_; Sigma-Aldrich) and sodium hydroxide (NaOH; Junsei Chemical) were used as received. Hydrous ruthenium oxide nanoparticles templated by double hydrophilic block copolymer shell (*h-*RuO_2_@PEO-*b*-PAA) were synthesized as reported previously^9^. Briefly, 25.1 mg PEO-*b*-PAA (equivalent to 0.20 mmol of carboxylic acid groups) was dissolved in 50.0 mL of deionized water under vigorous stirring until the solution was completely transparent. 0.10 mL of 4.0 M NaOH (0.40 mmol, 2 equivalents to carboxylic acid groups in PAA block) and then 17.8 mg (0.10 mmol) RuCl_3_·*x*H_2_O were introduced to the solution. Subsequently, 0.10 mL of 10.0 M hydrazine (1.0 mmol) was added to the resulting suspensions under vigorous stirring. After a few seconds, the solution color became dark cyan. The resulting solution was dialyzed against deionized water using a dialysis membrane (MWCO 12000–14000; SpectraPore) to remove residuals. The prepared *h-*RuO_2_@PEO-*b*-PAA solution exhibited fairly high colloidal stability, which lasted more than one year without any precipitation. Dry powder of *h-*RuO_2_@PEO-*b*-PAA was obtained by using a rotary evaporator. RuO_2_@C was obtained by heating the dried *h-*RuO_2_@PEO-*b*-PAA at the rate of 10 °C min^−1^ to an annealing temperature and then annealing in air at 400 °C for 2 h.

### Characterization

The morphology and size of RuO_2_@C were investigated by using transmission electron microscopy (TEM; JEOL, JEM-2100F; accelerating voltage at 200 kV with Gatan CCD camera). The functional groups of RuO_2_@C annealed at different temperatures were analysed by X-ray photoelectron spectroscopy (XPS; Thermo Fisher, K-alpha). The crystallography of RuO_2_ was investigated by high power X-ray diffractometer (XRD; Rigaku, D/MAX 2500 V/PC).

### Catalyst inks

Catalyst inks were prepared by mixing 4 mg catalyst composite in a mixture of 50 μl of 0.05% Nafion solution (Sigma-Aldrich) and 450 μl of ethanol by sonication for 30 min. 6 μl of the ink was transferred onto the 4 mm-diameter glassy carbon (GC) disk electrode of Pt/GC ring/disk electrode (ALS) and then dried at ambient temperature. Our RuO_2_@C was compared with *ah-*RuO_2_ (agglomerates of 30~50 nm primary particles, Sigma-Aldrich) as the more anhydrous control and *h-*RuO_2_ (Alfa Aesar) as the more hydrous control. Both controls were used as received. The catalyst composites were prepared by mixing the catalysts with 20 wt. % Ketjen Black 600 as a conducting agent. Pt/C (20 wt % loading of Pt on carbon black, Alfa Aesar) was also used as the catalyst composite for comparison.

### Electrochemistry

The electrocatalytic activity and stability of the catalysts were measured by using rotating ring disk electrode (RRDE; ALS) and potentiostat (Bio-Logic, VMP3). The catalyst-coated RRDE as a working electrode was immersed in a glass cell containing 0.1 M KOH. Hg/HgO (XR400, Radiometer Analytical) and Pt wire were used as reference and counter electrodes, respectively. All the potentials were reported in V_RHE_ (V versus RHE; RHE = reversible hydrogen electrode) in this work even if the potential values were read from potentiostats in V_Hg/HgO_: V_RHE_ = V_Hg/HgO_ + 0.93 V in 0.1 M KOH (aq). The ORR polarization voltammograms at 10 mV s^−1^ were obtained in the O_2_-saturated electrolyte between +0.2 V_Hg/HgO_ and −0.8 V_Hg/HgO_ at various rotation speed (400, 900, 1600 or 2500 rpm). At the same time, +0.4 V_Hg/HgO_ was applied to the ring electrode of RRDE to detect peroxide formed from the disk electrode by oxidizing the peroxide completely. Pure faradaic currents were reported in this work by subtracting background capacitive currents obtained in N_2_-saturated electrolyte from the overall reduction currents from disk electrodes. The collection efficiency (N) was estimated at 0.42 (the same as the theoretical value) in 10 mM potassium ferricyanide (K_3_Fe(CN)_6_) in 0.1 M KOH electrolyte under Ar atmosphere. The number of electrons transfer (n) of ORR was calculated by using: n = 4 |*I*_*d*_|/(|*I*_*d*_| + *I*_*r*_/N) where ${I}_{d}$ and ${I}_{r}$ are the disk and ring currents, respectively. The OER polarization voltammograms at 10 mV s^−1^ were obtained in the N_2_-saturated electrolyte between +0.35 V_Hg/HgO_ and +0.9 V_Hg/HgO_ at 1600 rpm. Mass-transfer-corrected currents (i_K_) were used for Tafel plots: i_K_ = i i_L_/(i_L_ − i) with i_L_ = limiting current^22^.

### Zn–air battery

Zn–air cells were constructed in a previously reported configuration^23^ based on: Zn plate (Alfa Aesar) as an anode, carbon on nickel mesh (MEET, Korea) as a gas diffusion layer (GDL) of an cathode, microporous membrane (Celgard 3501) as a separator and Ni mesh as current collectors with 6 M KOH aqueous electrolyte. 100 μl catalyst ink was loaded on a carbon GDL electrode (geometric area = 2.834 cm^2^) and the catalyst-loaded electrode was dried at 80 °C for >1 h. Zn-air cells were galvanostatically discharged and charged at various currents by a potentiostat (Bio-Logic, VMP3).

## Results and Discussion

**Figure 1:**
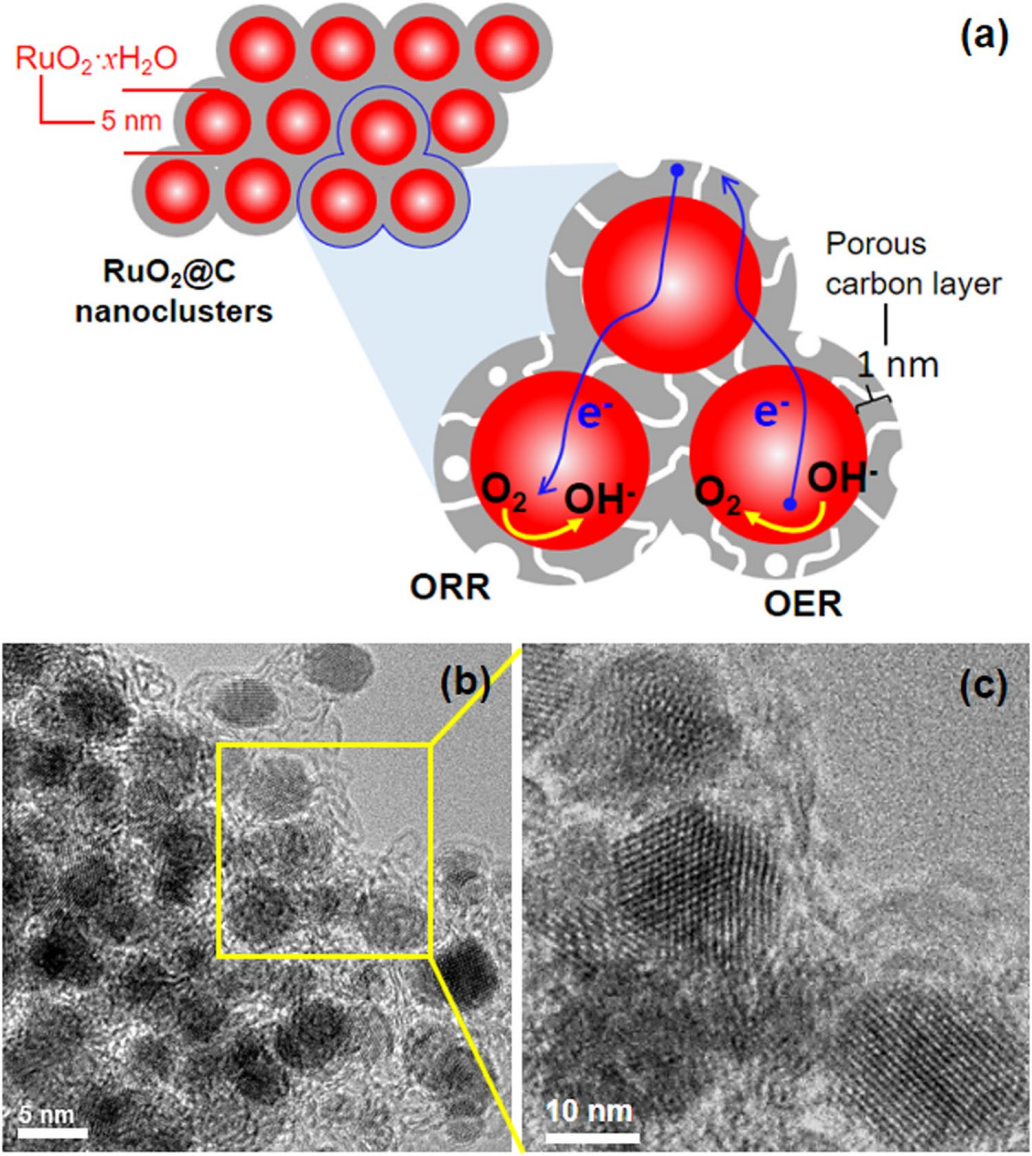
RuO_2_@C nanoclusters. (**a**) Schematic. (**b** and **c**) TEM images.

The ruthenium precursors templated by PEO-*b*-PAA were converted to RuO_2_ nanoparticles (4–5 nm in size) embedded in a continuous carbon matrix phase forming RuO_2_@C nanoclusters (Figs. 1 and S1). The electrostatic interaction between the ruthenium precursor cations and the anionic PAA blocks in the double hydrophilic block copolymer PEO-*b*-PAA afforded RuO_2_ spherical nanoparticles. The additional hydrophilic PEO blocks decorating the surface of the nanoparticles and exposed to solution stabilized the nanoparticles in solution, preventing them from aggregation.

Completely anhydrous form of RuO_2_ (*ah*-RuO_2_) has high crystallinity while its completely hydrous form (*h*-RuO_2_) is amorphous with no characteristic XRD patterns (Fig. S2). Partially hydrous RuO_2_’s (*x*-RuO_2_) show the in-between patterns depending on their hydration degree. RuO_2_@C showed a well-defined X-ray diffraction (XRD) RuO_2_ pattern at annealing temperatures higher than 350 °C. The amorphous characteristics of RuO_2_@PEO-*b*-PAA decreased with increasing temperature as its hydrated RuO_2_ was dehydrated with *x* decreasing in RuO_2_·*x*H_2_O. After annealing at 400 °C (i.e. the temperature used for preparing RuO_2_@C), the apparent crystallographic peaks of RuO_2_ were identified. The crystallite size calculated by Scherrer equation increased with the annealing temperature (i.e. 12 nm at 350 °C; <17 nm at 400 °C (RuO_2_@C); and <20 nm at 450 °C). The crystallite size of *x*-RuO_2_, obtained upon heating *h*-RuO_2_ at 400 °C, was 23 nm, which was larger than that of RuO_2_@C obtained by heating RuO_2_@PEO-*b*-PAA at 400 °C. *ah*-RuO_2_ showed the largest crystallite size among the materials studied (27 nm).

**Figure 2:**
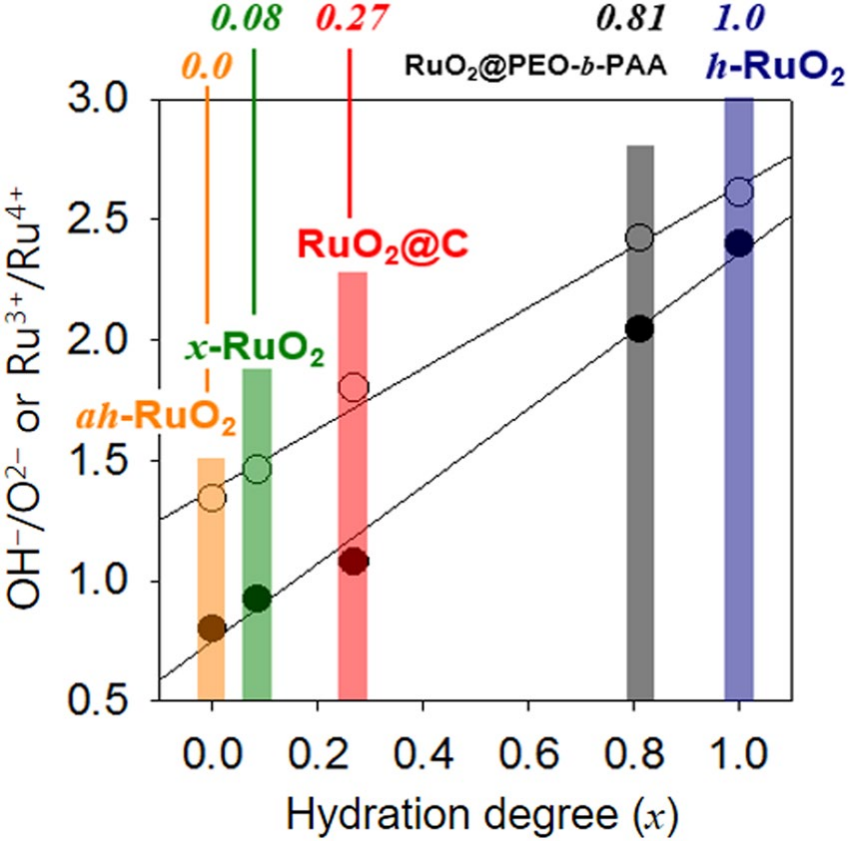
Hydration degree (*x*). The *x* values of *ah*-RuO_2_ and *h*-RuO_2_ were assumed to be 0.0 and 1.0, respectively. The OH^−^ to O^2−^ or Ru^3+^ to Ru^4+^ peak area ratios were used as a measure of the hydration degree. The *x* values of other RuO_2_ samples were estimated from the ratios obtained by interpolating two pre-fixed points of the XPS peak ratio versus *x*.

X-ray photoelectron spectra (XPS) measurements confirmed that the RuO_2_ crystallites in RuO_2_@C as well as the amorphous RuO_2_ phase in RuO_2_@PEO-*b*-PAA were both hydrated (Fig. S3a–d). Significant amounts of surface-adsorbed H_2_O and OH^−^ species were identified in the O1s spectra of both samples. Thus, surface water was dominant in RuO_2_@PEO-*b*-PAA, while OH^−^ prevailed in RuO_2_@C. In addition to lattice Ru(IV), Ru(III) species originating from hydrous Ru(III)–OH was found in the Ru3d spectra of both samples, although the relative amount of Ru(III) to Ru(IV) significantly decreased after thermal annealing^24^. When compared with *h*-RuO_2_ and *ah*-RuO_2_, the material prepared herein showed intermediate properties (Fig. S3e and f). Thus, the main peak in the O1s spectra of RuO_2_@C was placed between the surface OH^−^-characteristic peak of *h*-RuO_2_ and the lattice O^2−^-characteristic peak of *ah*-RuO_2_. The OH^−^ to O^2−^ or Ru(III) to Ru(IV) area ratios of RuO_2_@C (1.1 or 1.8, respectively) were between those of *ah*-RuO_2_ (0.4 or 1.3, respectively) and *h*-RuO_2_ (2.4 or 2.6, respectively). The relative hydration degree (*x*) of RuO_2_@C was estimated to be 0.27 (i.e. a quarter hydrous) by interpolating the peak ratio data as a measure of the hydration and assuming *x* values of 0.0 and 1.0 respectively for *ah*-RuO_2_ and *h*-RuO_2_ (Fig. 2). The *x* values of RuO_2_@PEO-*b*-PAA and *x*-RuO_2_ were 0.81 (i.e. highly hydrated) and 0.08 (nearly anhydrous), respectively. Therefore, we can consider RuO_2_@C to be formed by a mixture of hydrous and anhydrous RuO_2_ phases^25–27^. The anhydrous characteristics were dominant in the bulk properties (e.g., crystallography), whereas the hydrous characteristics were relevant when considering the surface properties (e.g., XPS spectra).

When used as an electrocatalyst, the structure of RuO_2_@C is expected to be beneficial owing to several reasons. First, the nanosized catalyst provided a large electrocatalytic surface area per mass. This high surface area was achieved by using the PEO-*b*-PAA template, which restricted the growth of RuO_2_ primary particles during the synthesis. Second, the electrons effectively reached the catalyst surface through the continuous carbon phase of the RuO_2_@C nanoclusters. Third, non-carbon PEO-*b*-PAA residues generated voids and pore space after carbonization, thereby allowing reactants to be readily transferred through the porous carbon matrix. Fourth, the carbon matrix surrounding RuO_2_ possibly prevented the catalyst from dissolution.

**Figure 3:**
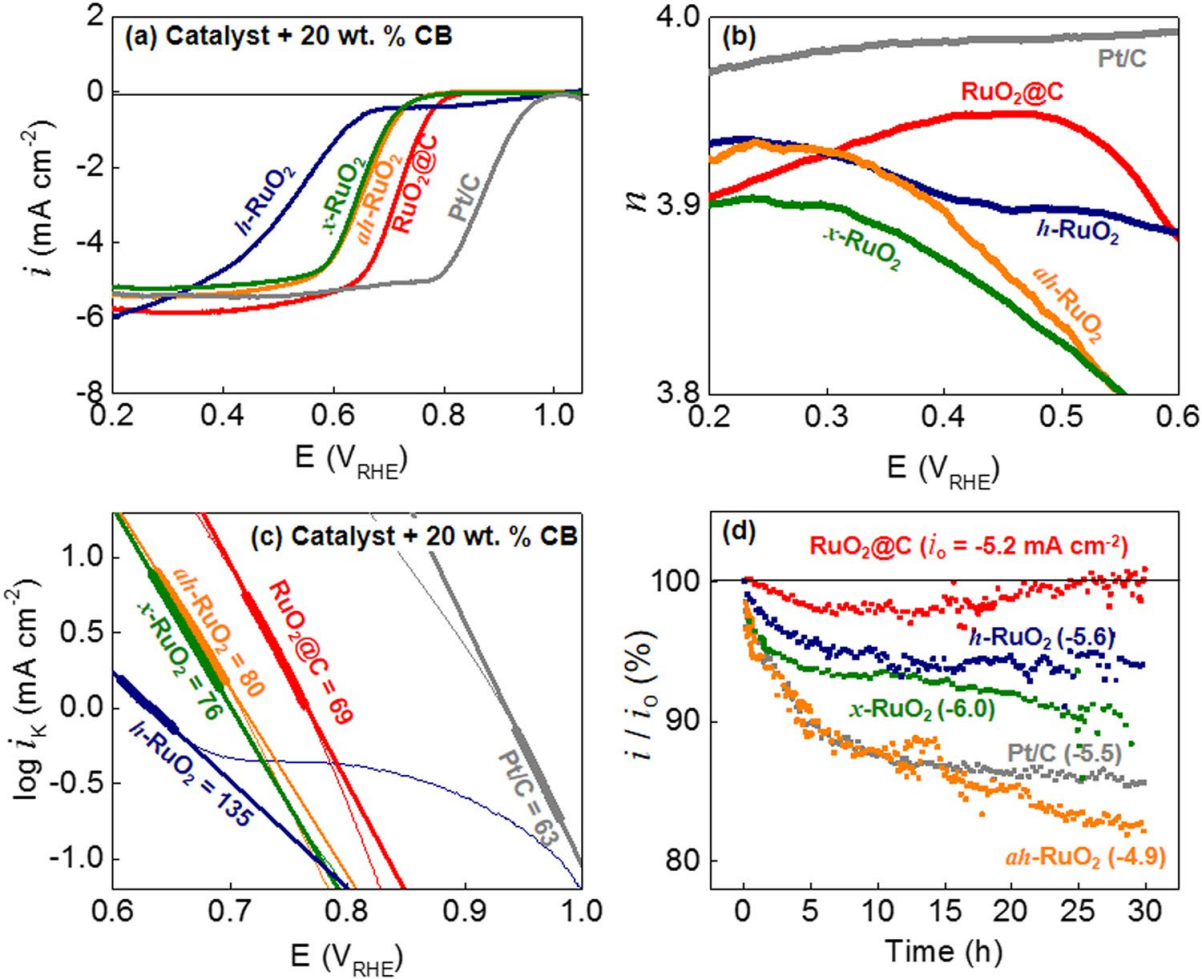
ORR in O_2_-saturated 0.1 M KOH (aq). (**a**) ORR polarization curves at 1,600 rpm and 10 mV s^−1^. (**b**) Electron transfer number (*n*). (**c**) Tafel plots. Mass transfer-corrected currents (*i*_K_) are used. Tafel slopes (b) are indicated in mV dec^−1^. (**d**) Chronoamperometric stability of ORR at +0.4 V_RHE_. The initial currents are indicated next to the names of the catalysts.

While ORR electroactivity of RuO_2_ has been rarely reported, its OER and hydrogen evolution reaction (HER) activities have been widely investigated^28^. Poor electroactivities (i.e. low ORR currents and high overpotentials) have been reported for RuO_2_ (Table S1, ESI†)^3, 29–31^. As an example, RuO_2_ showed a potential at half of the limiting current (i.e. *E*_2/L_ at *i*_L_/2) of +0.56 V_RHE_ at −1.2 mA cm^−2^ (cf. +0.9 V_RHE_ at −3 mA cm^−2^ for Pt/C)^29^. This high overpotential of RuO_2_ was indicative of very sluggish ORR kinetics. More seriously, the electron transfer number (*n*) was estimated to be ca. 2 (4 is the preferred value for *n*; discussed below). Interestingly, significantly higher ORR electroactivities were obtained herein even for a commercially available *ah*-RuO_2_ control sample (Fig. 3). The RuO_2_@C nanoclusters prepared herein, mixed at 20 wt% with carbon black (CB) as a conducting agent (RuO_2_@C in Fig. 3 and RuO_2_@C + CB in Fig. S4), showed the most rapid kinetics (*E*_2/L_ = +0.7 V_RHE_ at −3 mA cm^−2^) among the RuO_2_ ORR catalysts tested. When CB was not used, the onset potential was significantly shifted to negative potentials (RuO_2_@C in Fig. S4).

When compared with *h*-RuO_2_ and *ah*-RuO_2_, RuO_2_@C combined the advantages of both forms of RuO_2_: the RuO_2_@C was anhydrous-like in terms of smaller ORR onset overpotential; and hydrous-like due to its higher number of electron transfer. In the conductive environments realized by 20 wt. % CB, the presence of *ah*-RuO_2_ was beneficial in terms of the onset potential for ORR polarization (Fig. 3a). On the other hand, *h*-RuO_2_ was beneficial in terms of the number of electron transferred (*n*), especially at low overpotentials (Fig. 3b). Annealing at 400 °C (*x*-RuO_2_ in Fig. 3) allowed reducing the high overpotential of *h*-RuO_2_ while shifting the onset potential to that of *ah*-RuO_2_. However, the annealing promoted dehydration, decreasing the *n* values in *x*-RuO_2_ and reaching those of *ah*-RuO_2_. RuO_2_@C showed lower overpotentials as compared with the two extreme control samples (i.e. *h*-RuO_2_ and *ah*-RuO_2_) with *n* values comparable to those of *h*-RuO_2_. The hydration degree (*x* in RuO_2_·*x*H_2_O) of RuO_2_@C was higher than that of *x*-RuO_2_ (as revealed via XRD and XPS data in Figs. S2 and S3, respectively) such that high *n* values (>3.9) were obtained. The *n* values measured from disk and ring RRDE currents coincided with the values estimated from the Koutecky–Levich plots (Fig. S5). The 4e ORR showed by RuO_2_@C revealed complete reduction of oxygen without producing hydrogen peroxide (i.e. the 2e ORR intermediate product). RuO_2_@C showed a Tafel slope at low overpotentials of 69 mV dec^−1^, close to that of Pt/C (Fig. 3c). Therefore, we concluded that the carbon matrix compensated the poor conductivity of *h-*RuO_2_, preventing the Ohmic potential shift (i.e. low overpotential as in the case of conductive *ah*-RuO_2_) and enabling efficient utilization of the active mass (i.e. high *n* values as in the case of *h*-RuO_2_).

The chronoamperometric ORR stability of RuO_2_ was also improved by the carbon shell in RuO_2_@C (Fig. 3d). The currents of *ah*-RuO_2_ and Pt/C at +0.4 V_RHE_ significantly decreased (to 80% of the initial values) after 30 h. In contrast, RuO_2_@C showed excellent stability without current decay. Although Pt/C is the best ORR catalyst from the kinetic standpoint, this material is well known to suffer from instability as a result of Pt aggregation via surface diffusion and dissolution/re-precipitation processes.

**Figure 4:**
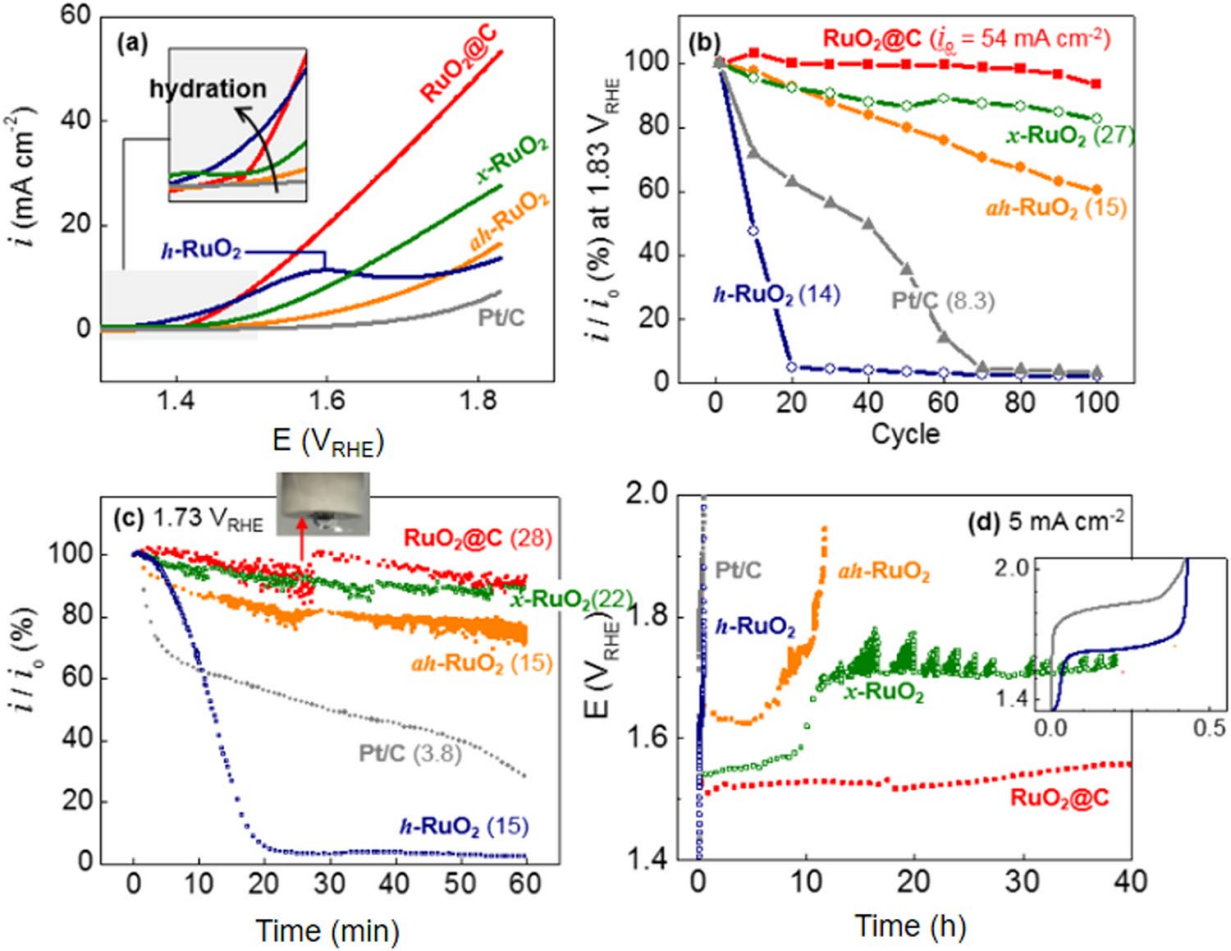
OER in N_2_-saturated 0.1 M KOH (aq). (**a**) OER polarization curves at 1,600 rpm for the first potential sweep cycles at 10 mV s^−1^. (**b**) OER current retention at 1.83 V_RHE_ during repeated cyclic voltammograms. (**c**) Chronoamperometric stability of OER at 1.73 V_RHE_ for 60 min in 0.1 M KOH. The initial currents are indicated next to the names of the catalysts. (**d**) Chronopotentiometric stability of OER at 5 mA cm^−2^ for 40 h.

The OER electroactivities of RuO_2_@C were investigated with the full knowledge that RuO_2_ is one of the best OER catalysts^32^. RuO_2_@C, regardless of CB presence, showed remarkably higher current densities and clearly reduced overpotentials as compared to its noncarbon-matrix counterpart (*ah*-RuO_2_; Figs. 4a and S6; current at 1.8 V_RHE_ = 54 mA cm^−2^ (RuO_2_@C with CB, >31 mA cm^−2^ (RuO_2_@C without CB), >13 mA cm^−2^ (*ah*-RuO_2_ with and without CB) and >5.4 mA cm^−2^ (Pt/C); potential at 10 mA cm^−2^ (*E*_10_) = 1.52 V_RHE_ (RuO_2_@C with CB), <1.59 V_RHE_ (RuO_2_@C without CB), <1.75 V_RHE_ (RuO_2_ with and without CB) and <1.87 V_RHE_ (Pt/C)). To the best of our knowledge, the RuO_2_@C material prepared herein showed better OER current and onset potential values than any previously reported RuO_2_ catalyst (Table S1). Note that all polarization data obtained herein were not IR-compensated unless specified. Since IR compensation correction seriously affected the OER polarization (not the ORR), the percentage of compensation (*f*) should be carefully selected (Fig. S7): *R*_c_ = *f R*_u_, where *R*_c_ is the resistance for correction and *R*_u_ is the uncompensated resistance between the working and reference electrodes. The *E*_10_ of RuO_2_@C significantly decreased from 1.55 V_RHE_ at *f* = 0% to 1.48 V_RHE_ at *f* = 85% and 1.47 V_RHE_ at *f* = 100%. The nanosized particles of the catalyst well connected to electric and ionic pathways would be partly responsible for the improved OER electroactivity of RuO_2_@C. The hydrated nature of RuO_2_ can also account for the improved results. Thus, the OER onset potentials decreased with increasing hydrous character of RuO_2_ in the catalysts (Fig. 4a, inset) as follows: *ah*-RuO_2_ < *x*-RuO_2_ < RuO_2_@C < *h*-RuO_2_.

Despite its good onset potential characteristics, *h*-RuO_2_ showed important stability issues (Fig. 4b). This OER instability has been previously ascribed to RuO_2_ dissolution issues during OER, especially in the case of *h*-RuO_2_^7, 33^. A broad anodic peak was found at 1.6 V_RHE_ for *h*-RuO_2_ during the initial anodic scan of the potential (Fig. 4a). The stability of *h*-RuO_2_ was improved by the carbon matrix in RuO_2_@C. Thus, the specific current density of RuO_2_@C reached 54 mA cm^−2^ at 1.83 V_RHE_ and slightly decreased (to 93% of the initial value) after repeating the potential sweep between 1.3 V_RHE_ and 1.83 V_RHE_ 100 times at 10 mV s^−1^ (Fig. 4b). The carbon matrix of RuO_2_@C was believed to protect its partially hydrated RuO_2_ from dissolution. RuO_2_@C also showed high stability under different chronoamperometric conditions (1.73 V_RHE_ for 60 min, 0.1 M KOH) (Fig. 4c). The noise-like fluctuation in the current during OER was produced by O_2_ bubbles generated on the electrode surface. The RuO_2_ particle size in the carbon matrix of RuO_2_@C did not significantly change after the chronoamperometric test, in contrast with Pt/C that showed particle agglomeration under identical conditions (Fig. S9).

Carbon corrosion is one of the most serious issues of air electrodes in Zn–air batteries during rechargeable operations. The loss of solid carbon via corrosion leads to catalyst loss and electrode leakage, thereby resulting in performance decay^34^. Carbon corrosion processes can be described as follows^35, 36:^

1 $${\rm{C}}+2{{\rm{H}}}_{2}{\rm{O}}\to {{\rm{CO}}}_{2}+4{{\rm{H}}}^{+}+4{{\rm{e}}}^{-}\quad {\rm{E}}=1.034{{\rm{V}}}_{{\rm{RHE}}}$$

2 $${\rm{C}}+{{\rm{H}}}_{2}{\rm{O}}\to {\rm{CO}}+2{{\rm{H}}}^{+}+2{{\rm{e}}}^{-}\quad {\rm{E}}=1.345{{\rm{V}}}_{{\rm{RHE}}}$$

3 $${\rm{CO}}+{{\rm{H}}}_{2}{{\rm{O}}}^{-}\to {{\rm{CO}}}_{2}+2{{\rm{H}}}^{+}+2{{\rm{e}}}^{-}\quad {\rm{E}}=0.724{{\rm{V}}}_{{\rm{RHE}}}$$

When considering the reduction potentials, carbon corrosion is thermodynamically inevitable in the OER potential range. The only way of mitigating carbon corrosion is the reduction of the OER overpotential. The overpotential advantage of RuO_2_@C was clearly reflected in the OER stability (i.e. the potential remained stable below 1.6 V_RHE_ at 5 mA cm^−2^ over 40 h, Fig. 4d). In contrast, the rest of RuO_2_ samples significantly increased their overpotential values with time at the same current density.

**Figure 5:**
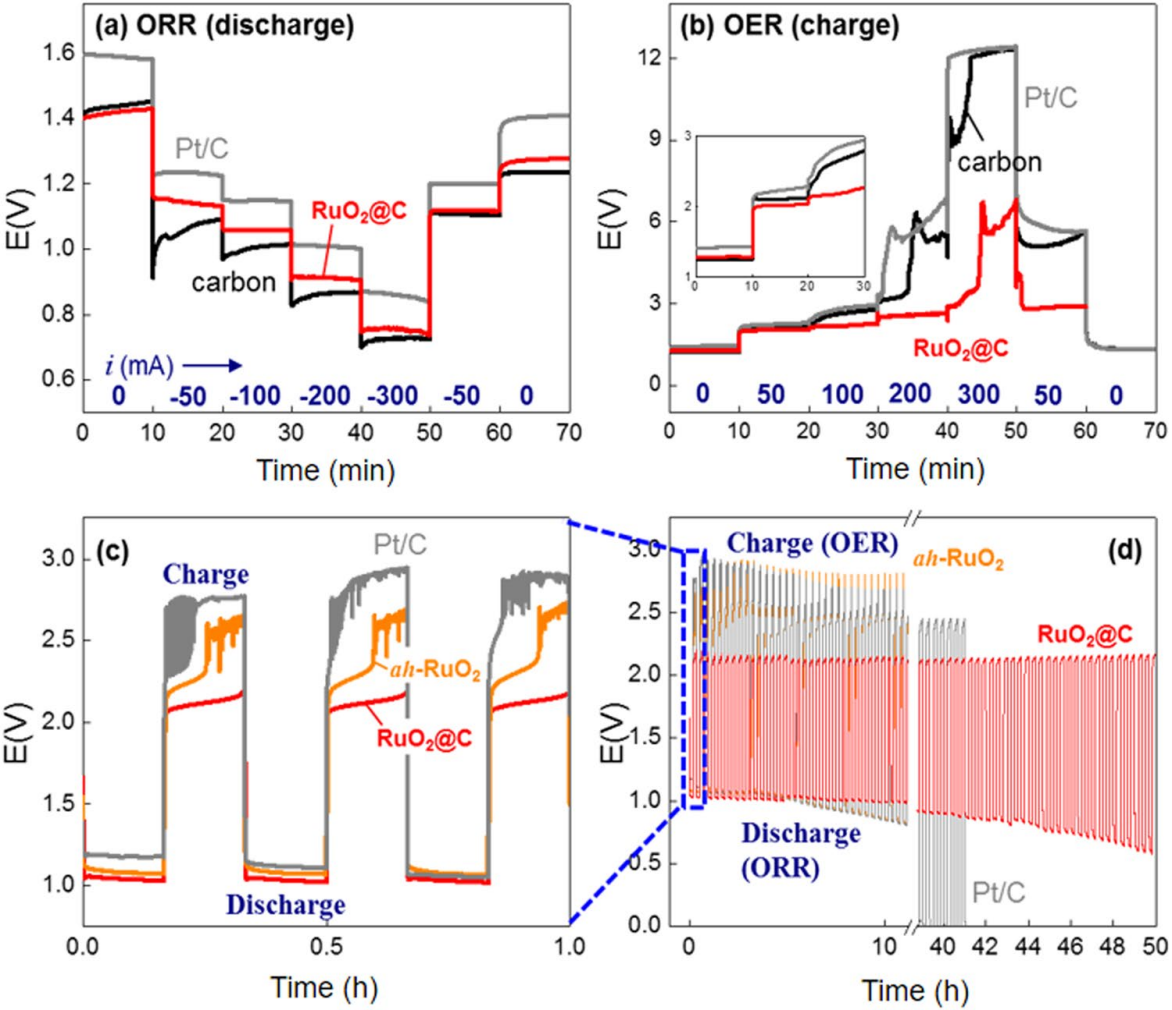
Zn–air batteries. 20 wt% carbon black used for RuO_2_@C. Pt loading of Pt/C is 20 wt%. (**a**) Discharge rate capability at 20 mA cm^−2^ charge. ORR proceeds on air electrodes. The currents used for discharge are indicated in mA (geometric electrode area = 2.8 cm^2^). (**b**) Charge rate capability at 20 mA cm^−2^ discharge. OER proceeds on air electrodes. The currents used for charge are indicated in mA (geometric electrode area = 2.8 cm^2^). (**c** and **d**) Galvanostatic charge/discharge cycles at 20 mA cm^−2^.

Reversible operation of Zn–air batteries (Fig. S10a) was achieved by using the bifunctional RuO_2_@C material (Fig. 5). RuO_2_@C, Pt/C or a carbon electrode were used as air electrodes, while zinc was selected as the metal electrode. The ORR and OER kinetics were investigated from the discharge and charge rate capabilities under fixed slow charge (Fig. 5a) and discharge (Fig. 5b) conditions, respectively. Pt/C showed the lowest ORR overpotential, although it developed a severe OER overpotential (even larger than the non-catalytic carbon electrode) especially at high current densities (>200 mA). RuO_2_@C showed a very good ORR polarization behaviour despite presenting larger overpotentials than Pt/C. Interestingly, the overpotential difference between RuO_2_@C and Pt/C in the Zn–air battery cells (ca. 0.1 V) was lower than the half wave potential difference (E_L/2_) between them in the linear sweep voltammograms (ca. 0.2 V, Fig. 3a). The RuO_2_@C-based battery was successfully charged at a lower potential as compared to Pt/C- and carbon electrode-based batteries, especially at high currents (Fig. 5b). Stable potential profiles were obtained in the presence of RuO_2_@C, up to fast charges of 200 mA. The potential difference between charge and discharge (Δ*E*_OER-ORR_) of RuO_2_@C (1.1 V) was significantly enhanced as compared to those of Pt/C and carbon air electrodes (1.6–1.7 V, Fig. S10b) at 100 mA (1.65 V for RuO_2_@C versus ca. 5 V for the rest of electrodes at 200 mA). The lower Δ*E*_OER-ORR_ value of RuO_2_@C was indicative of the higher ORR–OER reversibility of this material.

To further confirm the rechargeability of the RuO_2_@C-based Zn–air batteries, the cells were repeatedly discharged and charged in the galvanostatic manner at 20 mA cm^−2^ following a 20-min cycle period for 50 h (Fig. 5c and d). The potentials of RuO_2_@C remained stable at 1.04 V during discharge and at 2.11 V during charge for over 40 h or 120 cycles (Fig. S10c). The observed decrease in the ORR potential during discharge after 40 h was not caused by catalyst deterioration. Instead, this issue was caused by an electrolyte leakage through the gas diffusion layer of the air electrode, thereby hindering the oxygen supply. No problematic deterioration was found in the Zn–RuO_2_@C cell during OER operation. Unlike RuO_2_@C, Pt/C- or carbon-based Zn–air cells showed loss of performance during OER as a result of catalyst failure. Serious OER overpotentials were developed from the initial charge. When compared with the cell operation data previously reported, the RuO_2_@C-based cell demonstrated the most reversible behaviour among the published Zn–air cells^23, 37–40^. This cell showed a Δ*E*_OER-ORR_ value of 1.0 V, which is the lowest value among the air batteries reported (Fig. S10d and Table S2). The potential gap decreased to 0.85 V when operating the cell under a 100% oxygen stationary atmosphere instead of air. The reversibility of our Zn–oxygen cell was among the best reported so far, although lower Δ*E*_OER-ORR_ values have been reported in the literature (Fig. S11 and Table S2)^41–45^, although in these cells oxygen was forcibly introduced through the cells or electrolytes.

## Conclusions

The advantages of *h*-RuO_2_ and *ah*-RuO_2_ were combined by embedding partially hydrated RuO_2_ nanoparticles in a carbon matrix (RuO_2_@C). RuO_2_@C showed an *x* value of 0.27 (i.e. quarter hydrous). RuO_2_@C demonstrated both anhydrous- (i.e. lower ORR onset overpotential and superior OER stability) and hydrous-like (i.e. higher number of electron transfer during ORR and lower OER onset overpotential behaviour. The superior characteristics of RuO_2_@C were demonstrated by operating Zn–air battery cells. These cells showed the smallest overpotentials reported so far, thereby guarantying the efficient operation of rechargeable Zn–air batteries.

## Electronic supplementary material

Supplementary information
