# Supplementary material for: Bifunctional hydrous RuO2 nanocluster electrocatalyst embedded in carbon matrix for efficient and durable operation of rechargeable zinc–air batteries
Source: Sci Rep. 2017 Aug 2;7:7150. doi: 10.1038/s41598-017-07259-9 (PMC5540911; doi:10.1038/s41598-017-07259-9)
Supplement: Supplementary file 1 — Supplementary information [file 41598_2017_7259_MOESM1_ESM.pdf]

Supplementary information

Bifunctionl hydrous RuO_2 nanocluster electrocatalyst embedded in carbon matrix for efficient and durable operation of rechargeable zinc-air batteries

Han-Saem Park,[‡a] *Eunyong Seo,*[‡a] *Juchan Yang,*[a] *Yeongdae Lee,*[a] *Byeong-Su Kim**[a,b] *and Hyun-Kon Song**[a]

[a] School of Energy and Chemical Engineering, UNIST, Ulsan 44919, Korea.

[b] Department of Chemistry, UNIST, Ulsan 44919, Korea

[‡] These authors contributed equally to this work.

*Corresponding author: philiphobi@hotmail.com (H.-K. S.); bskim19@unist.ac.kr (B.-S. K.)

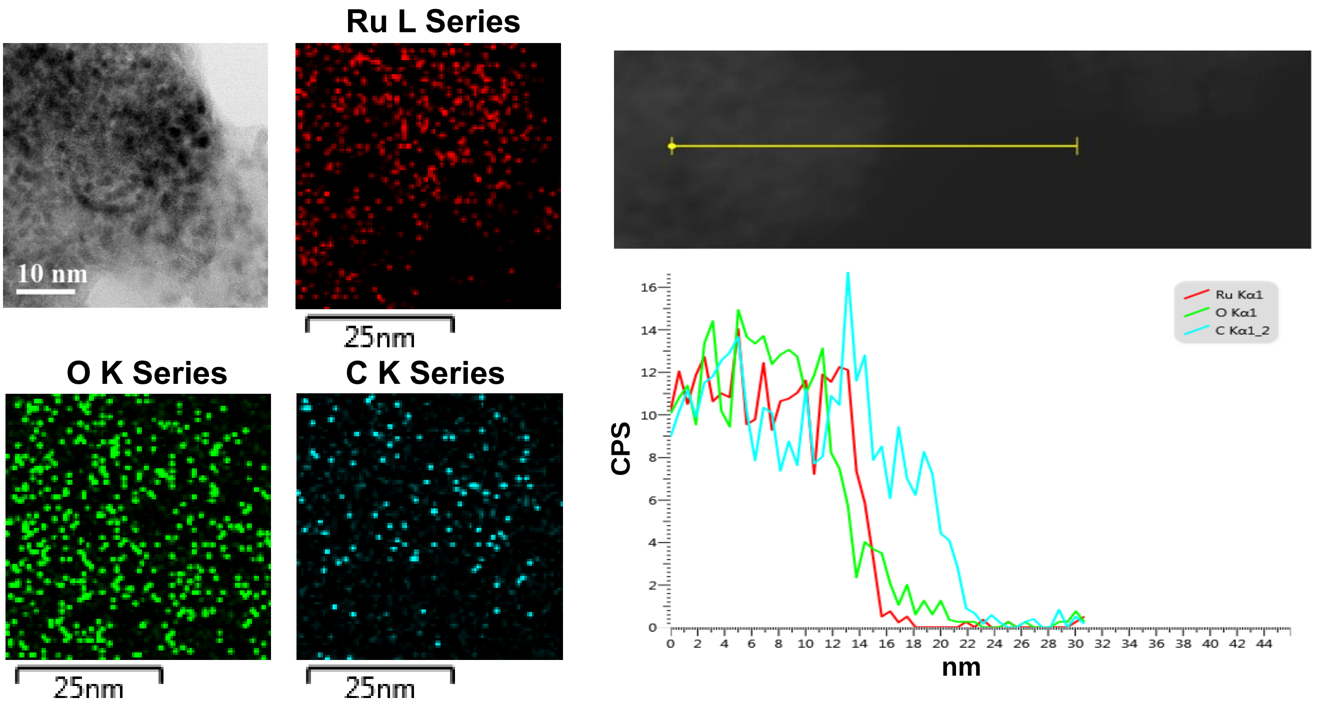

Figure S1. Scanning transmission electron microscopy (STEM) image, energy-dispersive X-ray spectroscopy (EDS) mapping of Ru, O and C and line mapping.

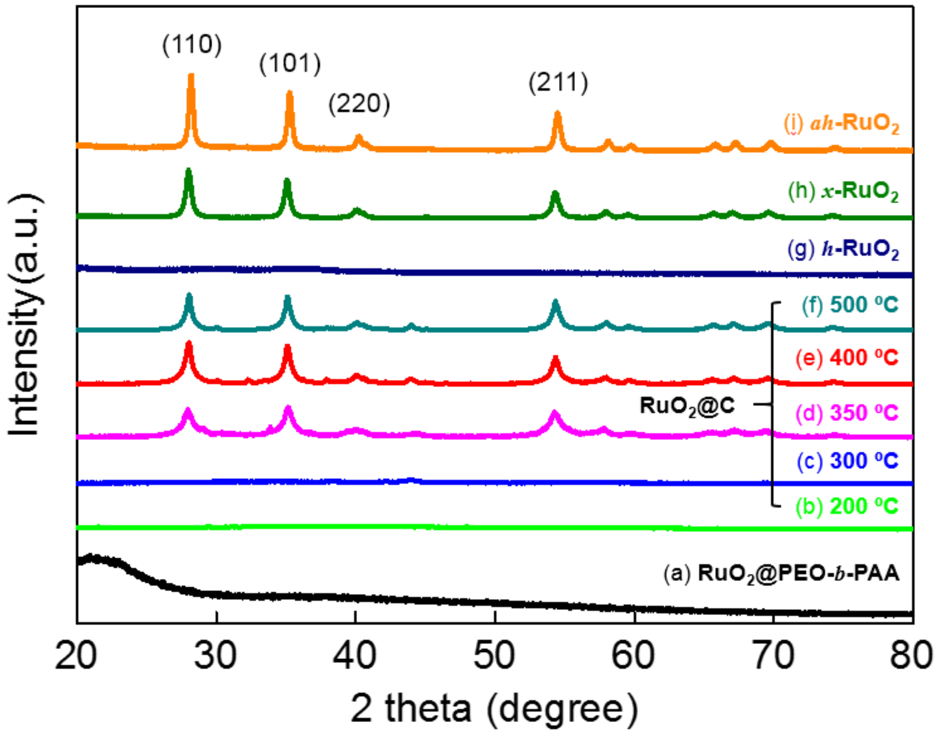

Figure S2. XRD patterns. (a) RuO_2@PEO-*b*-PAA. **(b to f)** RuO_2@C obtained after annealing RuO_2@PEO-*b*-PAA. The annealing temperatures were indicated. **(g)** *h*-RuO_2 = hydrous RuO_2 (x = high in $RuO_2 \cdot xH_2O$). **(h)** *x*-RuO_2 = *h*-RuO_2 thermally treated at 400 ºC. **(i)** *ah*-RuO_2 = anhydrous RuO_2 (x approaching 0 in $RuO_2 \cdot xH_2O$).

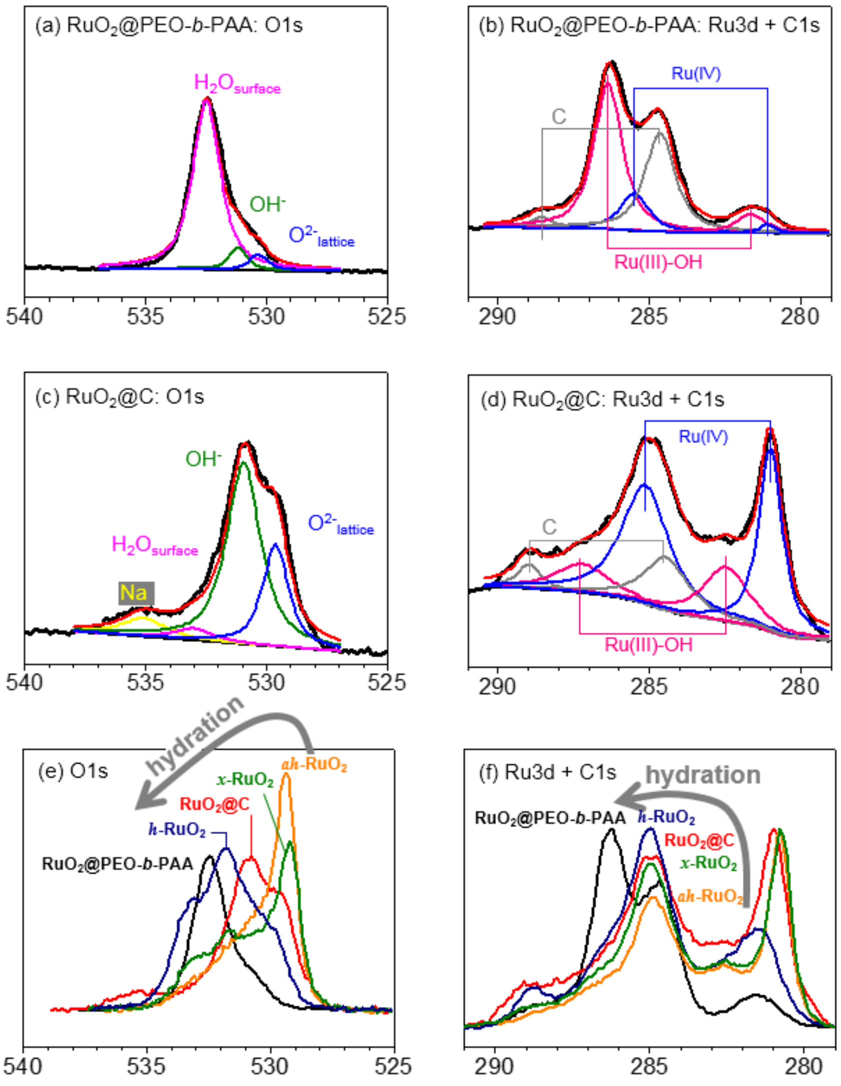

Figure S3. X-ray photoelectron spectra. Abscissa = binding energy (eV), ordinate = intensity in arbitrary unit. (**a** and **b**) O1s **(a)** and Ru3d + C1s **(b)** spectra of RuO_2@PEO-*b*-PAA after drying. (**c** and **d**) O1s **(c)** and Ru3d + C1s **(d)** spectra of RuO_2@C obtained by annealing RuO_2@PEO-*b*-PAA at 400 °C. 3 component spectra were used for deconvoluting O1s spectra: lattice oxygen (O^{2-}) at 530.4 eV; hydroxyl group (OH^-) at 531.2 eV; and surface-bound water (H_2O) at 532.5 eV. 6 component spectra were used for deconvoluting Ru3d and C1s spectra: Ru (IV) indicating RuO_2 at 281.0 and 285.5 eV; Ru(III) of hydrous Ru(III)-OH at 281.7 and 286.4 eV; C 1s at 284.6 and 288.5 eV. (**e** and **f**) O1s **(e)** and Ru3d + C1s **(f)** spectra of RuO_2@PEO-*b*-PAA, RuO_2@C, *ah*-RuO_2, *h*-RuO_2 and *x*-RuO_2.

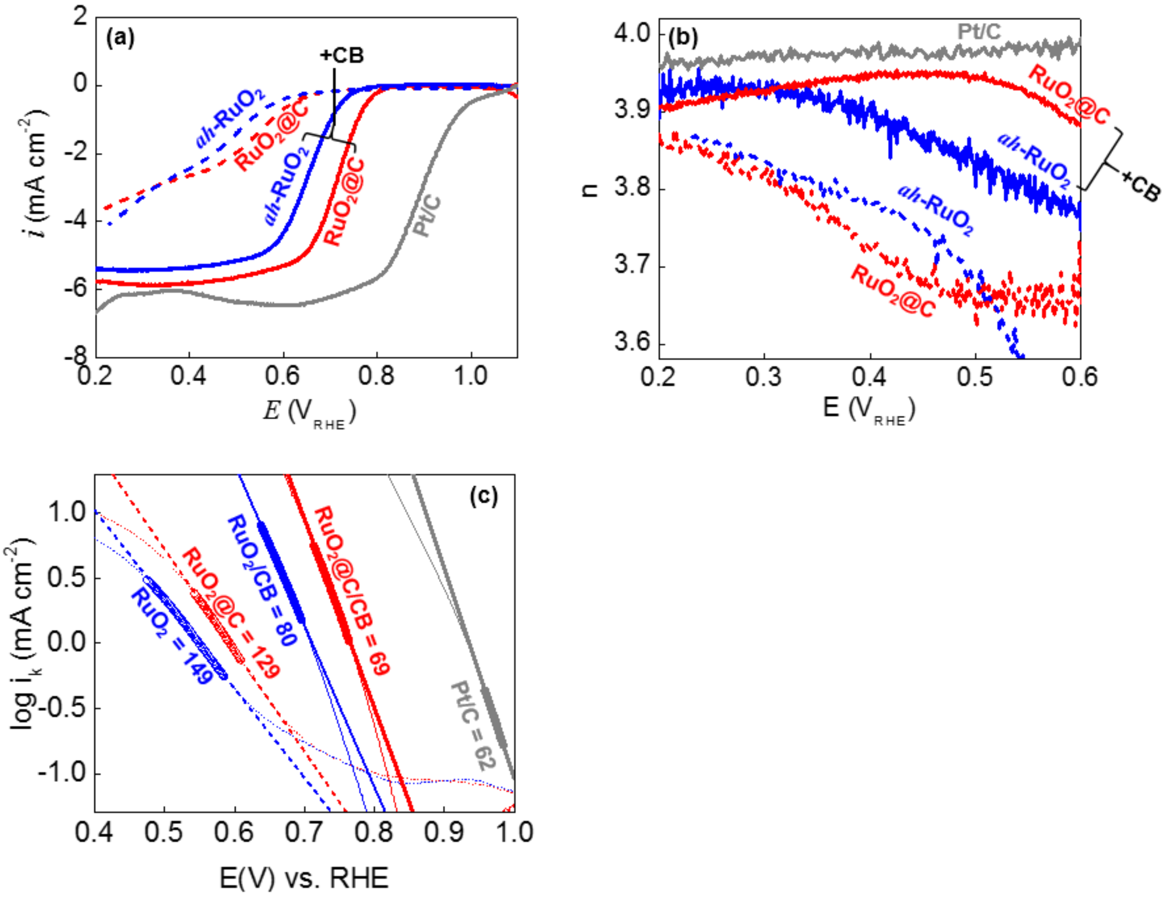

Figure S4. ORR in O_2-saturated 0.1 M KOH (aq). (a) ORR polarization curves at 1600 rpm and 10 mV s^{-1}. (b) Electron transfer number (*n*). (c) Tafel plots. Mass-transfer-corrected currents (i_K) were used. Tafel slopes (b) were indicated in mV dec^{-1}.

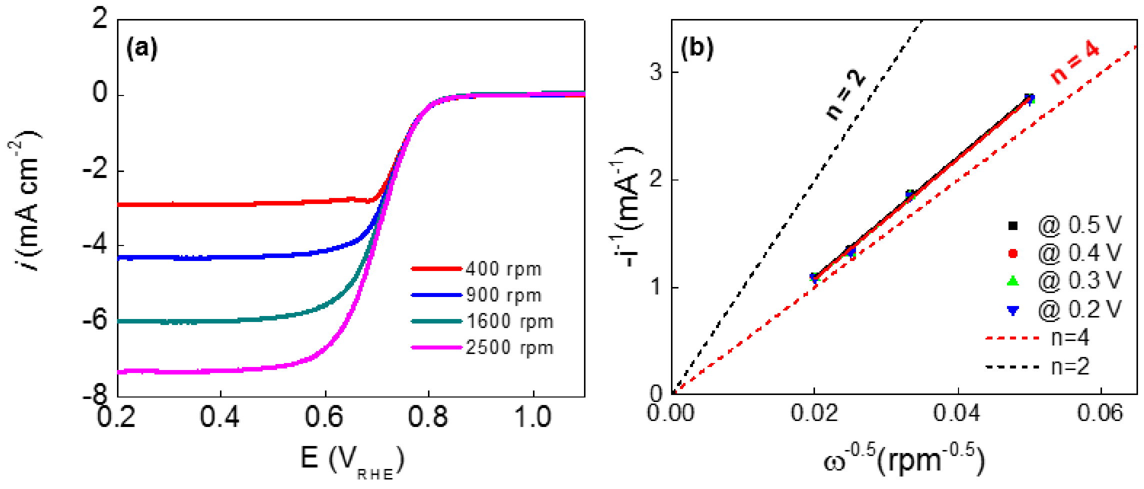

Figure S5. (a) ORR polarization curves of RuO_2@C/CB at various rotating speeds. **(b)** Koutecky-Levich plots of ORR on RuO_2@C/CB at various potentials. Dashed lines indicated the lines for two and four electron transfer cases (n = 2 and 4). The data for the dashed lines were calculated by Koutecky-Levich equation with the following values of parameters: bulk concentration of oxygen (C_O*) = 1.21 mol m^{-3}, diffusivity of oxygen (D_O) = 1.87×10^{-9} $m^2\cdot s^{-1}$ and kinematic viscosity (v) = 1×10^{-6} m^2 s^{-1}.

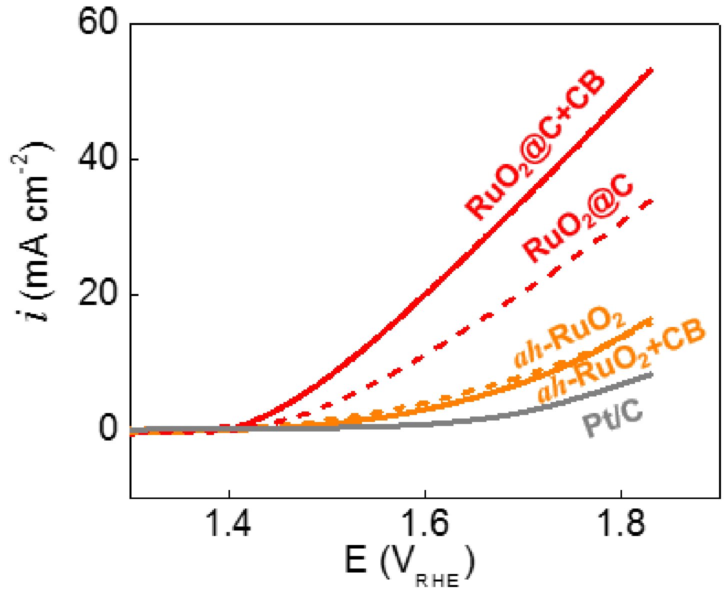

Figure S6. OER in N_2-saturated 0.1 M KOH (aq). OER polarization curves at 1600 rpm in the first potential sweep cycles at 10 mV s^{-1}.

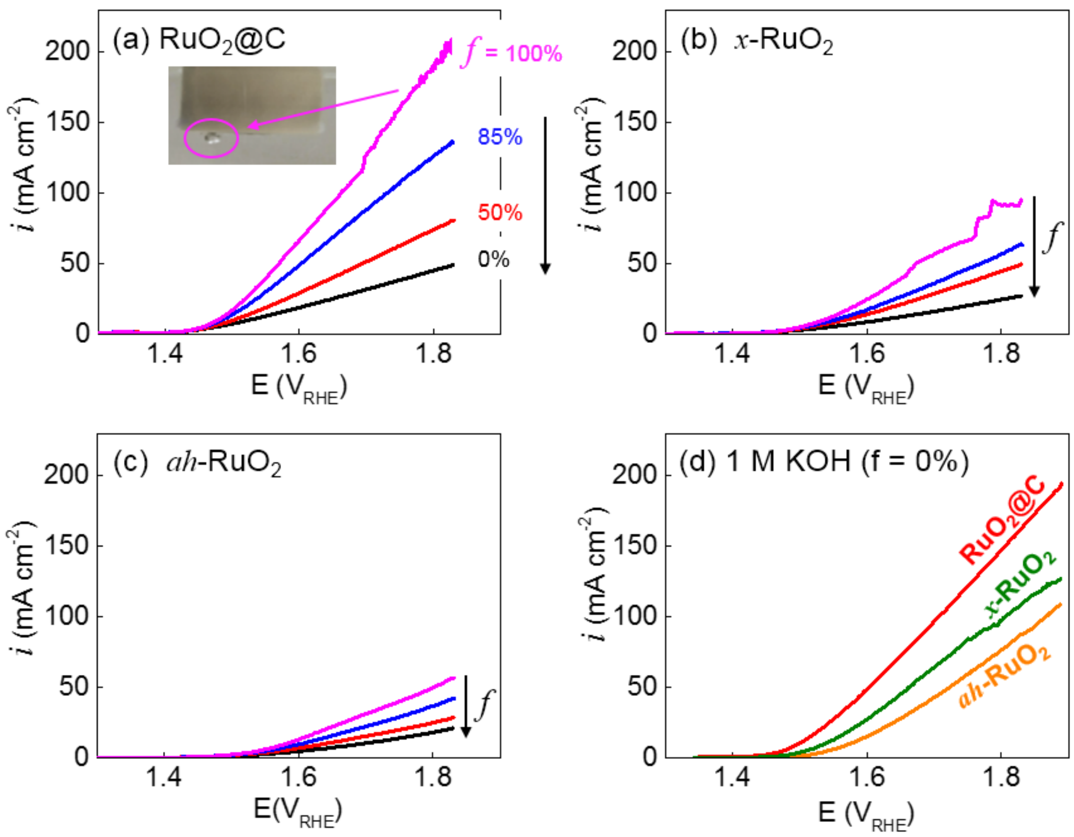

Figure S7. IR-compensated OER polarization at 10 mV s^{-1} on 1600 rpm. (**a** to **c**) 0.1 M KOH with various percentages of IR compensation *f*. (**d**) 1 M KOH at *f* = 0 %. The potential shift corrected by the IR compensation factor *f* affected the current values at a fixed potential and the onset potential seriously. Therefore, we should use the IR compensation function carefully and indicate the value of *f*.

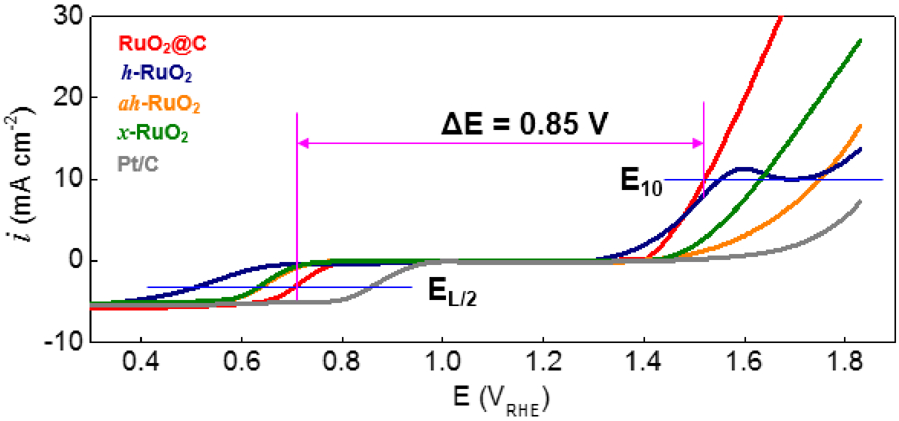

Figure S8. ORR and OER in 0.1 M KOH. The overall polarization curves of ORR and OER.

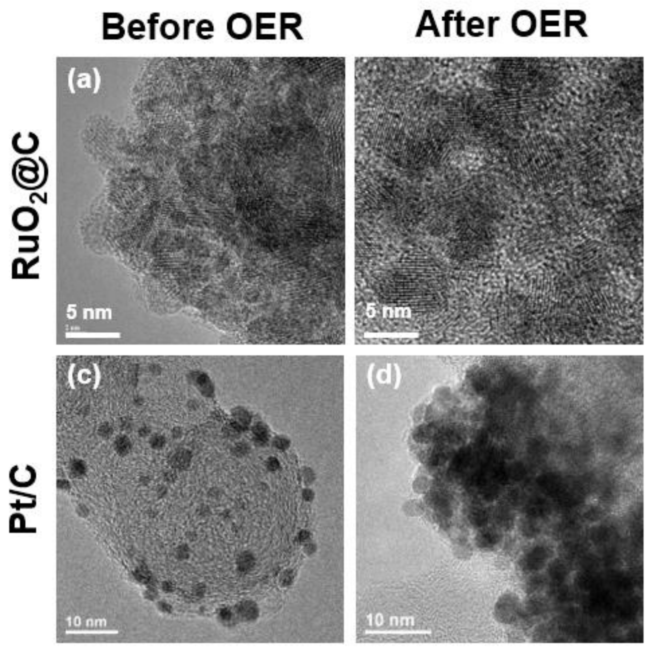

Figure S9. OER stability. (**a** to **d**) TEM images showing morphological changes of catalysts between before and after OER at 1.73 V_{RHE} for 1 h in 0.1 M KOH (aq).

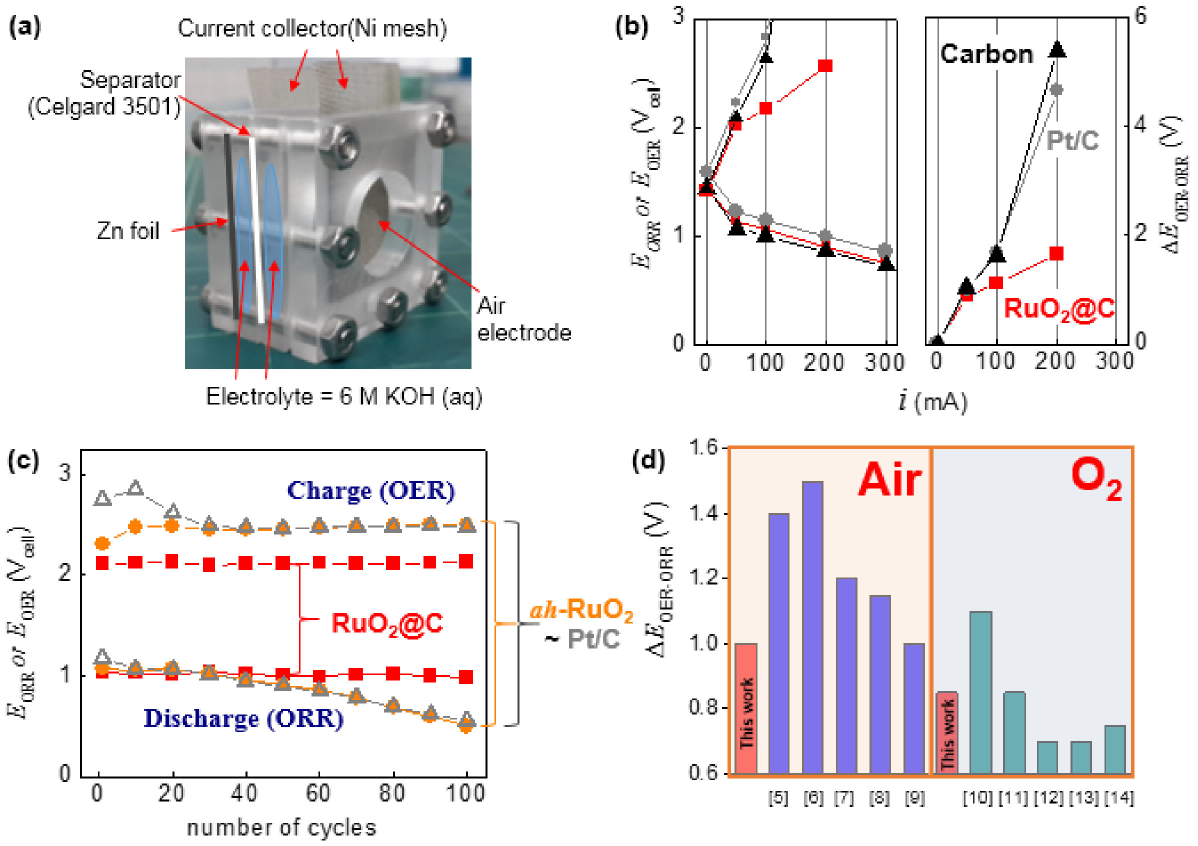

Figure S10. Zn-air battery cells. (a) The home-made Zn-air cell. Geometric area of air electrode = 2.2 cm^2. **(b)** The ORR and OER potentials (E_{ORR} or E_{OER} in the left panel) and the potential gaps between ORR and OER ($\Delta E_{OER\text{-}ORR}$) calculated from E_{ORR} and E_{OER} as a function of current. Data were taken from Figure 4a and b. **(c)** Cell potentials during discharge and charge. Refer to Figure 4c and d for its full potential profiles. **(d)** Comparison of $\Delta E_{OER\text{-}ORR}$ between RuO_2@C in this work and other electrocatalysts in other works [5-14]. The details were given in Table S2. The cells were categorized to groups in terms of fuels. 20 % and 100 % of molecular oxygen were used for Zn-air cells and Zn-O_2 cells, respectively.

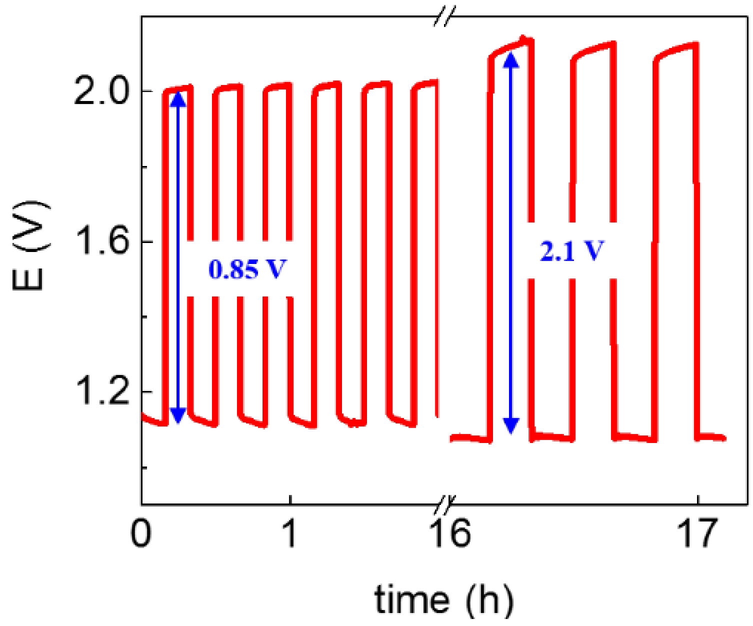

Figure S11. Galvanostatic charge/discharge cycles at 20 mA cm^{-2} in O_2 atmosphere at 1 atm.

Table S1. OER and ORR polarization.

Catalysts	ORR [a]			OER [b]	ΔE (V) [c]	Reference
	$E_{L/2}$ (V_{RHE})	$i_{L/2}$ (mA cm^{-2})	n	E_{10} (V_{RHE})		
Pt/C	0.90	-3.0	~4	-	-	This work
RuO_2@C	0.70	-3.0	3.8~3.9	1.55<br>1.48, 1.47*	0.85	This work
ah-RuO_2	0.65	-2.7	3.6~3.9	1.75<br>1.60, 1.58*	1.10	This work
RuO_2	0.56	-1.2	~2	1.76	1.20	[1]
RuO_2	0.40	-2.5	-	1.64	1.24	[2]
RuO_2	0.42	-1.5	-	1.60*	1.18	[3]
RuO_2	-	-	-	1.80	-	[4]

[a] $E_{L/2}$ = Half wave potential at the half of limiting current ($i_{L/2}$). [b] E_{10} = Potential obtained at +10 mA cm^{-2}. [c] ΔE = $|E_{L/2}$- $E_{10}|$. * Uncompensated IR was corrected in potentials. 85 % and 100 % compensation (f = 85 and 100 %) were used for the values with the asterisks in our work while the value of f was not reported in reference 3.

Table S2. Performance of rechargeable zinc-air batteries of various electrocatalysts published in the literature.

Catalyst	Charge (V) @ 20 mAcm^{-2}	Discharge (V) @ 20 mAcm^{-2}	Gap (V)	Cycle test condition	Atmosphere	Reference
RuO_2 nanoclusters embedded in carbon matrix	2.05	1.05	1.0	20min per cycle, 150 cycles, @ 20 mA/cm^2	Air	This work
$LaNiO_3$ macrospheres / N-CNT<br>N-CNT = nitrogen-doped carbon nanotube	2.4	1.0	1.4	10 min per cycle, 75 cycles @ 17.6 mA/cm^2	Air	[5]
MnO_2 nanotube / N-CNT	2.6	1.1	1.5	10 min per cycle, 50 cycles, @ 20 mA	Air	[6]
Co_3O_4 nanocrystals / N-CNT	2.2	1.0	1.2	10min per cycle, 240 cycles, @ 20 mA/cm^2	Air	[7]
Co_3O_4 nanoparticle / modified MnO_2 nanotubes	2.25	1.1	1.15	14 min per cycle, 60 cycles @ 15 mA/cm^2	Air	[8]
Co-doped TiO_2	2.1	1.1	1.0	20 h per cycle, 37 cycles @ 20 mA/cm^2	Not specified	[9]
RuO_2 nanoclusters embedded in carbon matrix	2.0	1.15	0.85	20min per cycle, 51 cycles, @ 20 mA/cm^2	O_2 atmosphere	This work
cobalt and nitrogen embedded carbon nanotubes	2.2	1.1	1.1	10 min per cycle, 16 cycles @ 15 mA/cm2	O_2 atmosphere	[10]
CoO / N-CNT + NiFe LDH<br>LDH = layered double hydroxide	2.05	1.2	0.85	240 min (4 hr) per cycle, 10 cycles @ 50.0 mA/cm^2	O_2 purged	[11]
$CoMn_2O_4$ nanoparticles anchored on N-doped rGO nanosheets	1.8	1.1	0.7	10 min per cycle, 100 cycles @ 20 mA/cm^2	O_2 flow	[12]
1D-$NiCo_2O_4$ nanostructures	1.8	1.1	0.7	40 min per cycle, 50 cycles @ 20.0 mA/cm^2	O_2 flow	[13]
$Co(II)_{1-x}Co(0)_{x/3}Mn(III)_{2x/3}S$ Nanoparticles / B/N-co-doped Mesoporous Nanocarbon	2.0	1.25	0.75	4 hr per cycle, 5 cycles @ 20 mA/cm^2	O_2 purged	[14]

References

1. Sunarso, J., Glushenkov, a. M., Torriero, a. a. J., Howlett, P. C., Chen, Y., MacFarlane, D. R. & Forsyth, M. Bi-Functional Water/Oxygen Electrocatalyst Based on PdO-RuO_2 Composites. *J. Electrochem. Soc.* 160, H74-H79 (2012).
2. Masa, J., Xia, W., Sinev, I., Zhao, A., Sun, Z., Grützke, S., Weide, P., Muhler, M. & Schuhmann, W. MnxOy/NC and CoxOy/NC nanoparticles embedded in a nitrogen-doped carbon matrix for high-performance bifunctional oxygen electrodes. *Angew. Chem. Int. Ed.* 53, 8508-8512 (2014).
3. Jiang, H., Yao, Y., Zhu, Y., Liu, Y., Su, Y., Yang, X. & Li, C. Iron Carbide Nanoparticles Encapsulated in Mesoporous Fe-N-Doped Graphene-Like Carbon Hybrids as Efficient Bifunctional Oxygen Electrocatalysts. *ACS Appl. Mater. Interfaces* 7, 21511-21520 (2015).
4. Zhang, J., Zhao, Z., Xia, Z. & Dai, L. A metal-free bifunctional electrocatalyst for oxygen reduction and oxygen evolution reactions. *Nat. Nanotechnol.* 10, 444-452 (2015).
5. Chen, Z., Yu, A., Higgins, D., Li, H., Wang, H. & Chen, Z. Highly Active and Durable Core–Corona Structured Bifunctional Catalyst for Rechargeable Metal–Air Battery Application. *Nano Lett.* 12, 1946-1952 (2012).
6. Chen, Z., Yu, A. P., Ahmed, R., Wang, H. J., Li, H. & Chen, Z. W. Manganese dioxide nanotube and nitrogen-doped carbon nanotube based composite bifunctional catalyst for rechargeable zinc-air battery. *Electrochim. Acta* 69, 295-300 (2012).
7. Lee, D. U., Park, M. G., Park, H. W., Seo, M. H., Wang, X. L. & Chen, Z. W. Highly Active and Durable Nanocrystal-Decorated Bifunctional Electrocatalyst for Rechargeable Zinc-Air Batteries. *Chemsuschem* 8, 3129-3138 (2015).
8. Du, G., Liu, X., Zong, Y., Hor, T. S. A., Yu, A. & Liu, Z. Co3O4 nanoparticle-modified MnO2 nanotube bifunctional oxygen cathode catalysts for rechargeable zinc-air batteries. *Nanoscale* 5, 4657-4661 (2013).
9. Han, L.-N., Lv, L.-B., Zhu, Q.-C., Wei, X., Li, X.-H. & Chen, J.-S. Ultra-durable two-electrode Zn-air secondary batteries based on bifunctional titania nanocatalysts: a Co2+ dopant boosts the electrochemical activity. *J. Mater. Chem. A* 4, 7841-7847 (2016).
10. Song, J., Zhu, C., Fu, S., Song, Y., Du, D. & Lin, Y. Optimization of cobalt/nitrogen embedded carbon nanotubes as an efficient bifunctional oxygen electrode for rechargeable zinc-air batteries. *J. Mater. Chem. A* 4, 4864-4870 (2016).
11. Li, Y., Gong, M., Liang, Y., Feng, J., Kim, J.-E., Wang, H., Hong, G., Zhang, B. & Dai, H. Advanced zinc-air batteries based on high-performance hybrid electrocatalysts. *Nat. Commun.* 4, 1805 (2013).
12. Prabu, M., Ramakrishnan, P. & Shanmugam, S. CoMn2O4 nanoparticles anchored on nitrogen-doped graphene nanosheets as bifunctional electrocatalyst for rechargeable zinc–air battery. *Electrochem. Commun.* 41, 59-63 (2014).
13. Prabu, M., Ketpang, K. & Shanmugam, S. Hierarchical nanostructured NiCo2O4 as an efficient bifunctional non-precious metal catalyst for rechargeable zinc-air batteries. *Nanoscale* 6, 3173-3181 (2014).
14. Wang, Z., Xiao, S., An, Y., Long, X., Zheng, X., Lu, X., Tong, Y. & Yang, S. Co(II)1–xCo(0)x/3Mn(III)2x/3S Nanoparticles Supported on B/N-Codoped Mesoporous Nanocarbon as a Bifunctional Electrocatalyst of Oxygen Reduction/Evolution for High-Performance Zinc-Air Batteries. *ACS Appl. Mater. Interfaces* 8, 13348-13359 (2016).
